# Neonatal hyperoxia exposure leads to developmental programming of cardiovascular and renal disease in adult rats

**DOI:** 10.1038/s41598-024-65844-1

**Published:** 2024-07-20

**Authors:** Marissa J. DeFreitas, Elaine L. Shelton, Augusto F. Schmidt, Sydne Ballengee, Runxia Tian, PingPing Chen, Mayank Sharma, Amanda Levine, Emily Davidovic Katz, Claudia Rojas, Carolyn L. Abitbol, Juanita Hunter, Shathiyah Kulandavelu, Shu Wu, Karen C. Young, Merline Benny

**Affiliations:** 1 Department of Pediatrics/Division of Nephrology, University of Miami Miller School of Medicine, Miami, FL USA; 2 Department of Pediatrics, Vanderbilt University Medical Center, Nashville, TN USA; 3 Department of Pediatrics/Division of Neonatology, Batchelor Children’s Research Institute, Miller School of Medicine, University of Miami, P.O. Box 016960 (R-131), Miami, FL 33101 USA; 4 Department of Pathology, Memorial Healthcare Systems, Hollywood, FL USA; 5 Department of Pediatrics/Division of Cardiology, University of Miami Miller School of Medicine, Miami, FL USA; 6 Interdisciplinary Stem Cell Institute, University of Miami Miller School of Medicine, Miami, FL USA

**Keywords:** Cardiovascular diseases, Kidney diseases

## Abstract

Premature infants are often exposed to hyperoxia. However, there is limited data regarding the mechanistic underpinnings linking neonatal hyperoxia exposure and its contribution to cardio-renal dysfunction in adults born preterm. Our objective was to determine whether neonatal hyperoxia induces systemic vascular stiffness and cardio-renal dysfunction in adulthood. Newborn rats were randomly assigned to room air (RA) or hyperoxia (85% O_2_) from postnatal day 1 to 14, then recovered in RA until 1 year of life. Arterial stiffness, cardio-renal histomorphometry, and fibrosis in the aorta, heart, and kidney were assessed. RNA-sequencing (RNA-seq) of the aorta and kidney was also done. Adult rats exposed to neonatal hyperoxia had increased aortic and mesenteric artery stiffness as demonstrated by wire and pressure myography. They also had cardiomyocyte hypertrophy, glomerulomegaly, and tubular injury. Hyperoxia exposure altered the transcriptome profile associated with fibrosis and matrix remodeling in the aorta and kidney. There was also increased TGF-β1 levels and fibrosis in the aorta, left ventricle, and kidney. In conclusion, neonatal hyperoxia exposure was associated with systemic vascular and cardio-renal alterations in 1-year-old rats. Further studies to determine how targeted therapies could reprogram cardio-renal injury after neonatal hyperoxia exposure are indicated.

## Introduction

Preterm-born individuals have a reduced lifespan despite improved neonatal survival^1^. Developmental programming refers to the ability of early life exposures to induce long-term alterations in organ function. Preterm morbidities and neonatal exposures are known to contribute to cardiovascular and renal dysfunction in later life^2–4^. Prematurity induces persistent pathology in the vascular tree, heart, and kidney due to an abrupt cessation of organogenesis coupled with various postnatal insults^5^. Neonatal hyperoxia exposure has been shown in clinical and experimental studies to be associated with bronchopulmonary dysplasia, retinopathy of prematurity, and kidney injury^6,7^. We have previously described the short-term association of neonatal hyperoxia with increased vascular stiffness, cardio-pulmonary dysfunction and renal injury in juvenile rats^8,9^. The long-term impact of neonatal hyperoxia exposure-induced bronchopulmonary dysplasia and pulmonary hypertension and its progression to cardio-pulmonary dysfunction has been described in children^10^. Transcriptome analysis has enhanced the ability to determine potential epigenetic changes and mechanisms driving the negative impact of neonatal hyperoxia on lung development in preterm infants. This has allowed for targeted therapeutic studies which have shown progress in attenuating the toxic effects of hyperoxia exposure on future cardio-pulmonary outcomes in experimental models^9^.

Very few studies have documented the long-term co-existing biomechanical changes in the systemic vasculature and kidney after neonatal hyperoxia exposure^11,12^. To our knowledge, no prior study has explored the impact of neonatal hyperoxia exposure on adult aortic and kidney differential gene expression profiles that could help guide the development of targeted therapies to reduce the burden of cardiovascular and renal dysfunction after preterm birth. Long-term kidney outcomes after neonatal hyperoxia exposure are limited and outcomes in rodents have generated inconsistent results ranging from decreased nephron number and glomerulomegaly to the absence of any structural abnormalities^11,13,14^.

Given that more preterm survivors are entering adulthood, we aimed to investigate the long-term effects of neonatal hyperoxia on the vasculature, heart, and kidney using the hyperoxia rodent model of prematurity.

Our objective was to determine whether neonatal hyperoxia exposure induces changes in systemic vascular compliance leading to cardio-renal pathology in adult rats. We hypothesized that neonatal hyperoxia exposure would be associated with vascular stiffness, cardiovascular and renal damage. The aims of our study were to: (1) investigate the long-term effects of neonatal hyperoxia on vascular compliance, (2) study the effects of hyperoxia delivered at the time of active postnatal nephrogenesis on glomerular and tubular integrity in adulthood, (3) evaluate whether the administration of neonatal hyperoxia promotes cardiovascular and renal fibrosis, and (4) assess how neonatal hyperoxia exposure alters gene expression in the aorta and kidney in adulthood.

## Results

### Effect of neonatal hyperoxia on body weight and kidney weight of adult rats

Neonatal rats (n = 26) were exposed to RA (n = 13) or hyperoxia (n = 13) (85% O_2_) for 2 weeks. They were then recovered in RA until they were 1-year-old. There was a 38% mortality in the pups exposed to hyperoxia when compared to the control group. Therefore, at 1 year, 13 rats (6 female and 7 male) in the RA group and 8 rats (2 female and 6 male) in the hyperoxia group were evaluated. Bodyweight trended higher in the hyperoxia group but did not achieve significance (Males: 806 ± 102 g vs 912 ± 99 g; RA vs O_2_ groups and Females: 394 ± 47 g vs 417 ± 154 g; RA vs O_2_ groups). The kidney weight was not significantly different between the RA and hyperoxia groups (males: 1.7 ± 0.3 g vs 1.6 ± 0.4 g; RA vs O_2_ groups and females: 1.6 ± 0.3 g vs 1.0 ± 0.3 g; RA vs O_2_ groups). The kidney/body weight ratio trended lower in the hyperoxia group however did not achieve significance (males: 2.2 ± 0.6 vs 1.8 ± 0.4; RA vs O_2_ groups and females: 4.5 ± 1.5 vs 2.6 ± 0.1; RA vs O_2_ groups).

### Neonatal hyperoxia is associated with increased vascular stiffness in adult rats

**Figure 1:**
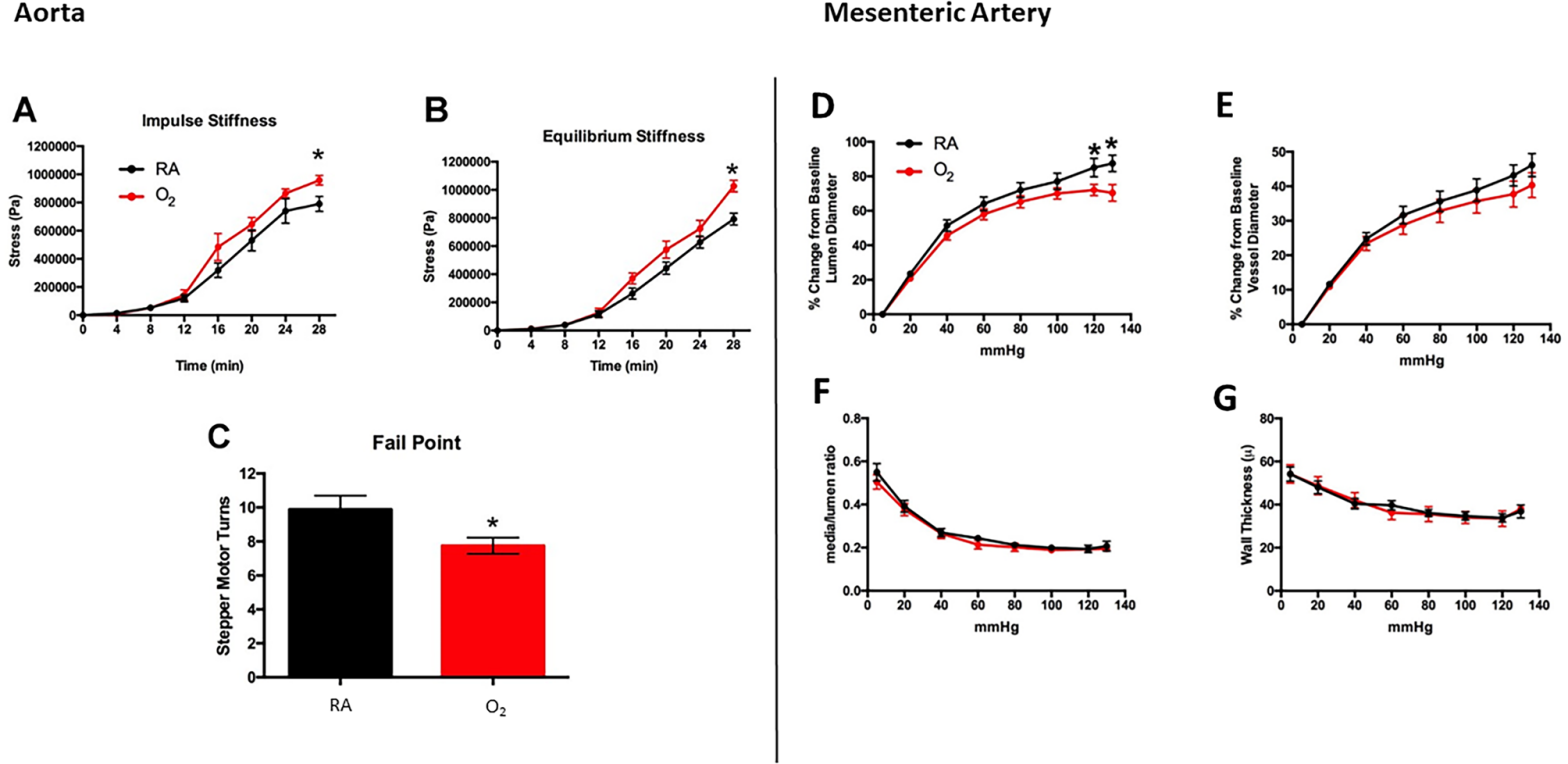
Neonatal hyperoxia is associated with systemic vascular stiffness in adult rats. Left panel showing assessment of vascular stiffness of the abdominal aorta in 1-year-old rats by wire myography. Neonatal hyperoxia increases (**A**) impulse stiffness (**B**) equilibrium stiffness and decreases (**C**) fail point in 1-year-old rats. n = 5–10/group. Right panel showing assessment of mesenteric arterial stiffness by pressure myography. (**D**) Neonatal hyperoxia exposed rats at 1 year had decreased change in lumen diameter from baseline. (**E**) Changes in baseline vessel diameter, (**F**) media/lumen ratio, and (**G**) vessel wall thickness were not different between the two group. n = 6–13/group. Data are mean ± SD; *p < 0.05; One way ANOVA for repeated measures with Bonferroni’s correction for multiple comparison were used for myography studies shown in A, B, D, E, F, and G. Unpaired, 2-tailed Student’s t-test was used for C. RA = room air; O_2_ = hyperoxia.

Pressure and wire myography are invaluable tools for studying the biomechanical properties of the vasculature. Wire myography shows increased impulse stiffness, equilibrium stiffness and fail point in the hyperoxia-exposed aortic arteries even after recovery in RA for 1 year (Fig. 1 Left panel, A–C). Exposure of neonatal rats to 2 weeks of hyperoxia resulted in a decreased mesenteric artery distensibility on pressure myography in adult rats, as seen by the decreased change in the lumen diameter from the baseline lumen diameter upon applying pressure (Fig. 1 Right panel, D–G). These findings suggest that prolonged neonatal hyperoxia exposure during the critical period of vascular development increases vascular stiffness in adult rats.

### Neonatal hyperoxia exposure is associated with cardiomyocyte hypertrophy, glomerulomegaly, and tubular injury in adult rats

**Figure 2:**
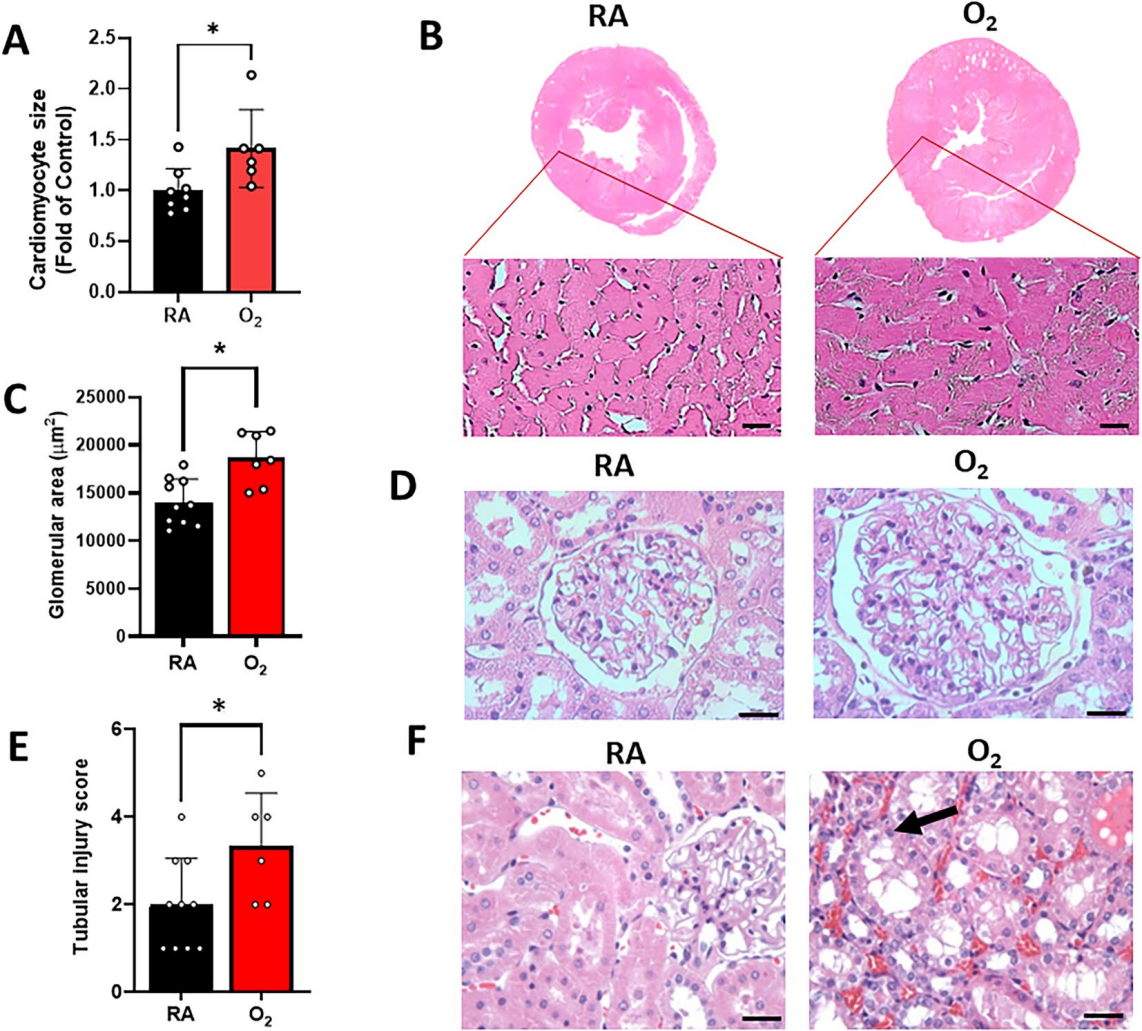
Neonatal hyperoxia exposure is associated with cardiomyocyte hypertrophy, glomerulomegaly, and tubular injury in adult rats. (**A**) Neonatal hyperoxia increased cardiomyocyte size at 1 year (p < 0.05; n = 6–8/group). (**B**) Hematoxylin and eosin-stained sections show cardiomyocyte hypertrophy after neonatal hyperoxia exposure (right) compared to RA (left) in 1-year-old rats. (**C**) Neonatal hyperoxia increased glomerular area (*p* < 0.05; n = 7–10/group). (**D**) Hematoxylin and eosin-stained kidney sections show glomerulomegaly after neonatal hyperoxia exposure (right) compared to RA (left). (**E**) Neonatal hyperoxia increased tubular injury scores (*p* < 0.05; n = 6–10/group). (**F**) Hematoxylin and eosin-stained kidney sections show tubular injury after neonatal hyperoxia exposure (right) compared to RA (left) in. Black arrow indicates vacuolization of tubular cell. Magnification is × 40. Scale bar is 50 μm. Data are mean ± SD; *p < 0.05 by unpaired, 2-tailed Student’s t-test; RA = room air; O_2_ = hyperoxia.

Rats exposed to neonatal hyperoxia developed cardiac hypertrophy as indicated by a 1.4-fold increased cardiomyocyte cross-sectional area (CSA; 916 ± 196 μm^2^ vs 1295 ± 349 μm^2^; RA vs O_2,_ *p* = 0.02; Fig. 2A, B). In addition, rats exposed to neonatal hyperoxia developed glomerulomegaly at 1 year as indicated by a significantly increased glomerular area (14,030 ± 2414 μm^2^ vs 18,660 ± 2737 μm^2^; RA vs O_2_, *p* = 0.01; Fig. 2C, D). The number of glomeruli per group was not different (12.2 ± 2.3 vs 12.7 ± 2.6 glomeruli/hpf, RA vs O_2_, *p* = 0.6). In addition, hyperoxia was associated with higher tubular injury scores at 1 year (2.00 ± 1.05 vs 3.33 ± 1.21; RA vs O_2_, *p* = 0.04; Fig. 2E, F). The injury was noted mainly in the proximal tubules. In addition, urine albumin (1134 ± 378 mcg/ml vs 1767 ± 915 mcg/ml; RA vs O_2_, *p* = 0.06) and urine neutrophil gelatinase-associated lipocalin, NGAL (249 ± 135 ng/ml vs 370 ± 380 ng/ml; RA vs O_2_, *p* = 0.22) trended higher while urine cystatin C trended lower (1485 ± 379 ng/ml vs 1008 ± 687 ng/ml; RA vs O_2_, *p* = 0.07) in the hyperoxia exposed group at 1 year, but these did not achieve significance.

### Neonatal hyperoxia causes long-term changes in the aorta and kidney transcriptome

**Figure 3:**
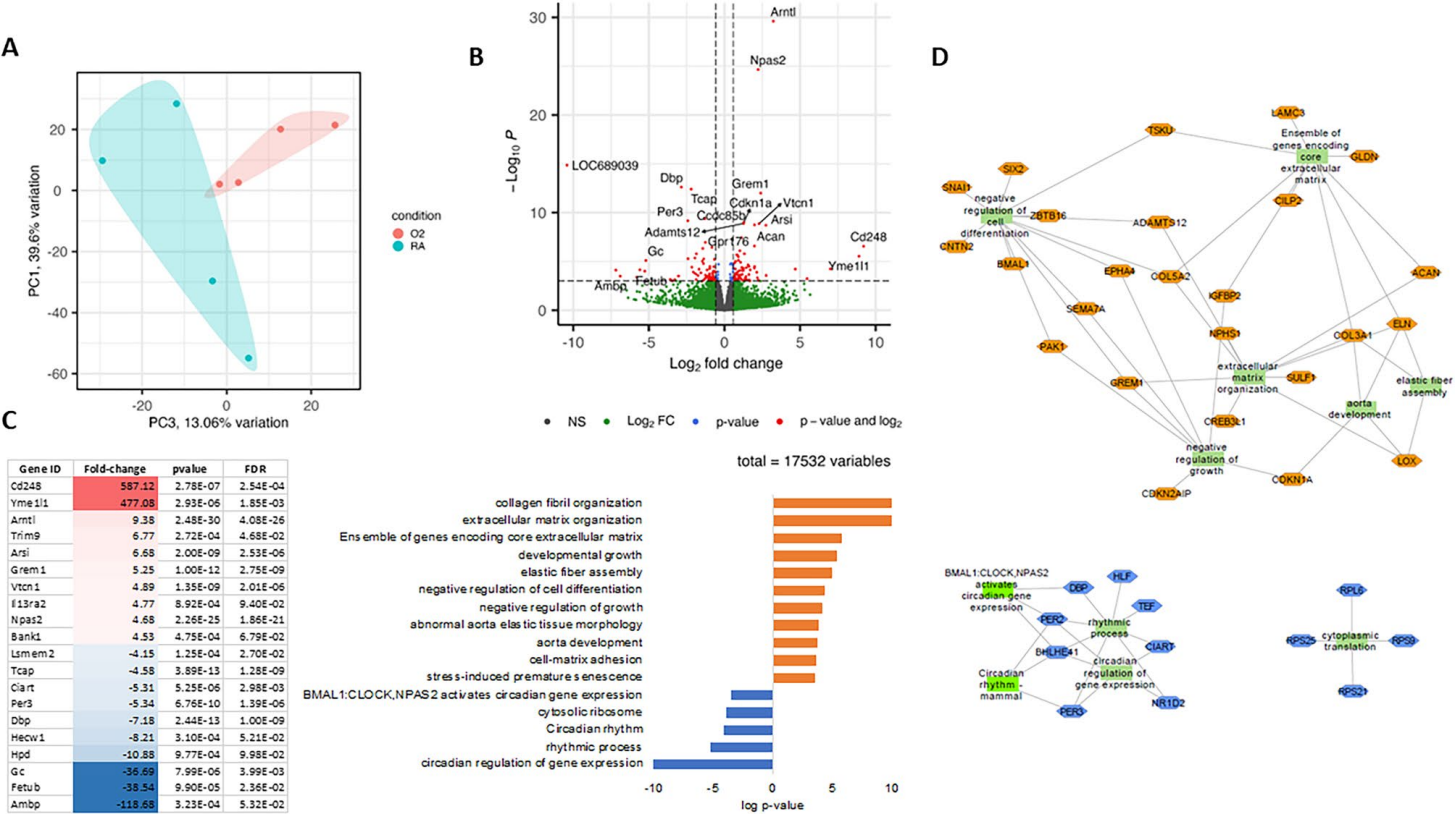
Neonatal hyperoxia exposure is associated with long-term transcriptional changes in adult rat aorta. (**A**) Neonatal hyperoxia resulted in persistent changes to the aorta’s transcriptome at 1 year with separation of RA and hyperoxia-exposed animals on principal component analysis. (**B**) Volcano plot showing differential gene expression in the aortas of RA-exposed compared to hyperoxia-exposed 1-year-old rats (fold change > 1.25 and FDR < 0.1). Red- significant. (**C, D**) Gene set enrichment analysis for Gene Ontology terms and Kegg pathways on ToppCluster showed that genes induced by hyperoxia in the aorta were associated with “collagen fibril organization”, “extracellular matrix organization”, “negative regulation of growth”, and “aorta development”. Genes suppressed by hyperoxia were associated with “circadian regulation of gene expression”, “circadian rhythm”, and “cytosolic ribosome”. n = 4/group, RA = room air; O_2_ = hyperoxia.

We used RNA-seq to identify signaling patterns altered by hyperoxia at 1 year. Hyperoxia resulted in changes to the aorta’s transcriptome with separation of RA and hyperoxia-exposed animals on principal component analysis (Fig. 3A). Volcano plot showed differential gene expression in the aortas of RA compared to hyperoxia-exposed 1-year-old rats (fold change > 1.25 and FDR < 0.1) (Fig. 3B). Hyperoxia differentially regulated 141 genes in the aorta, with 66 genes induced and 75 genes suppressed (Fig. 3C). Gene set enrichment analysis for Gene Ontology terms and Kegg pathways^15,16^ on ToppCluster showed that genes induced by hyperoxia in the aorta were associated with “collagen fibril organization”, “extracellular matrix (ECM) organization”, and “aorta development” (Fig. 3C, D). Genes suppressed by hyperoxia were associated with “circadian regulation of gene expression”, “circadian rhythm”, and “cytosolic ribosome” (Fig. 3C, D). These findings show that hyperoxia induces transcriptional signaling controlling the connective tissue and extracellular matrix in the aorta.

**Figure 4:**
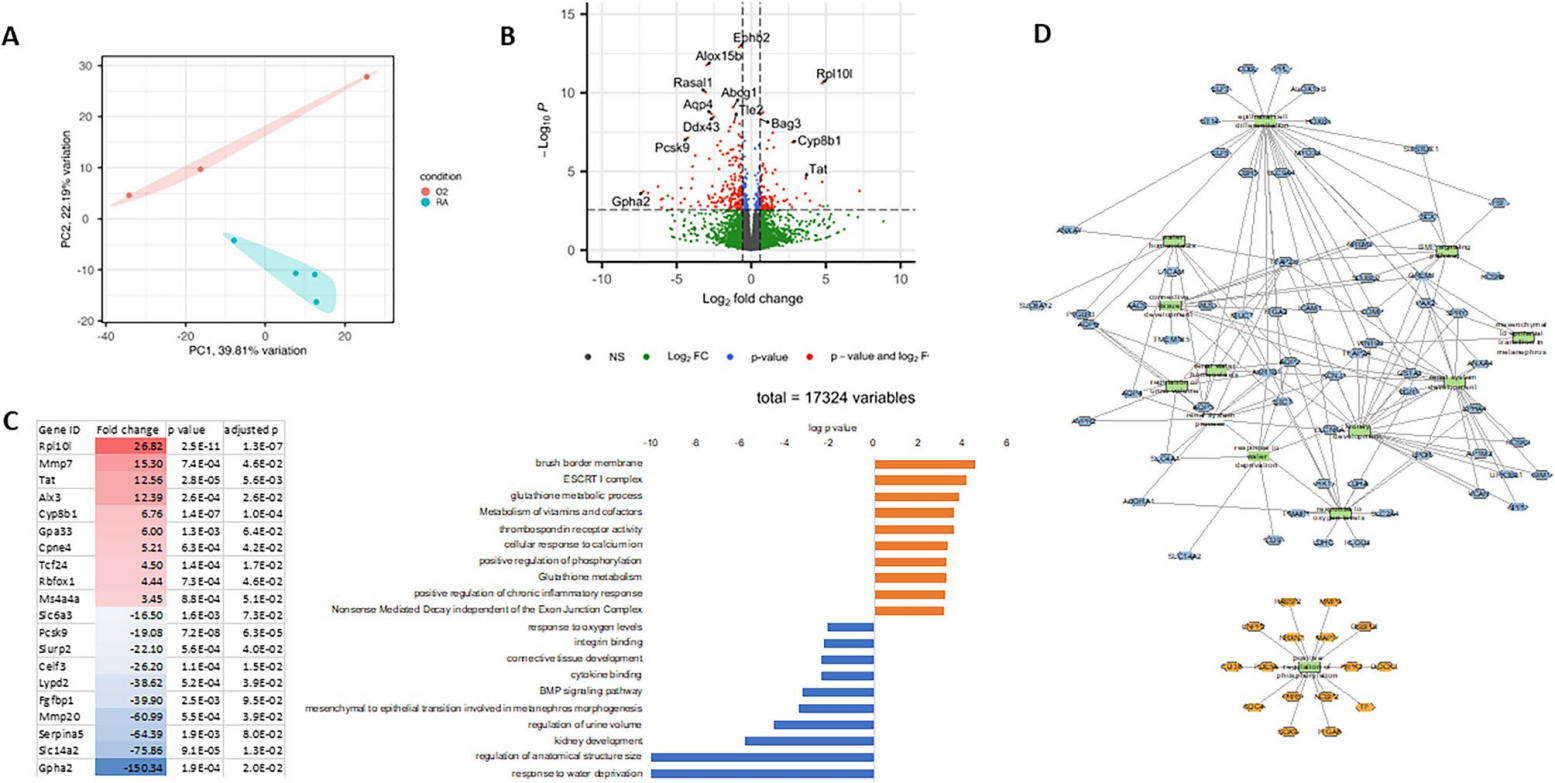
Neonatal hyperoxia exposure is associated with long-term transcriptional changes in the adult rat kidney. (**A**) Neonatal hyperoxia resulted in persistent changes to the kidney transcriptome at 1 year with clear separation of RA and hyperoxia-exposed animals by principal component analysis. (**B**) Volcano plot showing differential gene expression in the kidneys of RA-exposed compared to hyperoxia-exposed 1-year-old rats (fold change > 1.25 and FDR < 0.1). Red- significant. (**C, D**) Gene set enrichment analysis for Gene Ontology terms and Kegg pathways on ToppCluster showed that genes induced by hyperoxia were associated with “brush border” and “glutathione activity” and genes suppressed by hyperoxia were associated with “kidney development”, “connective tissue development”, “sodium ion transport”, “BMP signaling pathway”, “mesenchymal to epithelial transition involved in metanephros development” and “response to water deprivation”. Genes associated with the most relevant terms are shown in the network plot. n = 3–4/group, RA = room air; O_2_ = hyperoxia.

Similar to the aorta, hyperoxia resulted in persistent changes to the kidney transcriptome at 1 year (Fig. 4A). Volcano plot showed differential gene expression in the kidneys of RA compared to hyperoxia-exposed 1-year-old rats (fold change > 1.25 and FDR < 0.1) (Fig. 4B). Hyperoxia differentially regulated 426 genes in the kidney, with 161 genes induced and 265 genes suppressed (Fig. 4C). Gene set enrichment analysis for Gene Ontology terms and Kegg pathways on ToppCluster showed that genes induced by hyperoxia were associated with “brush border” and “glutathione activity” and genes suppressed by hyperoxia were associated with “kidney development”, “connective tissue development”, and “BMP signaling pathway”. (Fig. 4C). Genes associated with the most relevant terms are shown in the network plot (Fig. 4D) and show upregulation of matrix metalloproteinase 7 (MMP-7) involved in ECM deposition. These findings show that hyperoxia causes chronic changes in renal developmental pathways associated with nephron formation and connective tissue development.

**Figure 5:**
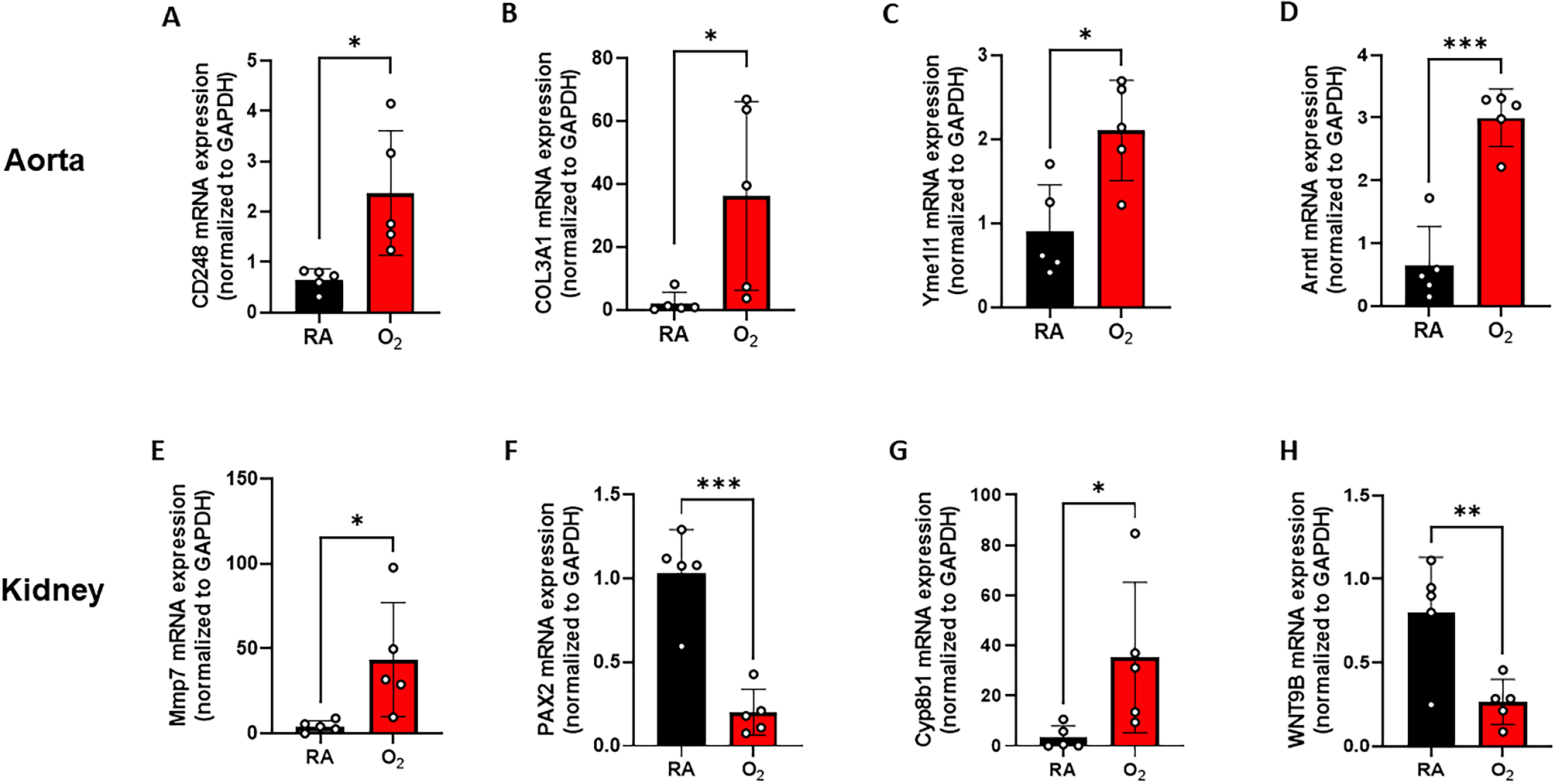
qRT-PCR validation of differentially regulated genes in the aorta and kidney of neonatal room air and hyperoxia exposed 1 year old rats. In the aortas of 1 year old rats, neonatal hyperoxia upregulated gene expressions of (**A**) CD248, (**B**) COL3A1**, **(**C**) Yme1l1, (**D**) Arntl. Hyperoxia upregulated gene expressions of (**E**) MMP7, (**F**) PAX2, (**G**) Cyp8b1, (**H**) WNT9B in the kidney of 1 year old rats. n = 5/group. Data are mean ± SD; *p < 0.05, **p < 0.01, ***p < 0.001 by unpaired, 2-tailed Student’s t-test; RA = room air; O_2_ = hyperoxia.

In addition, we performed qRT-PCR to verify select genes differentially regulated by hyperoxia. Neonatal hyperoxia was associated with increased mRNA expression of several genes involved in fibrosis, ECM organization and circadian rhythm such as CD248 (endosialin), collagen type 3 alpha 1 chain (COL3A1), aryl hydrocarbon receptor nuclear translocator-like protein 1 (Arntl), and YME1 like 1 ATPase (Yme1l1) in the aortas of 1-year-old-rats (Fig. 5A–D). CD248, a transmembrane glycoprotein that is expressed on fibroblasts and pericytes during tissue development, inflammation, and neovascularization, was the most differentially expressed gene between the RA and hyperoxia-exposed rat aortas at 1 year^17^. Neonatal hyperoxia was associated with altered mRNA expression of several genes involved in kidney development and fibrosis including matrix metallopeptidase (MMP7), paired box homeotic gene 2 (PAX2), wingless-type MMTV integration site family member 9B (WNT9B), and cytochrome P450 family 8 subfamily B member 1 (cyp8b1) at 1 year of age (Fig. 5E–H).

### Neonatal hyperoxia induces cardiac and renal fibrosis in adult rats

**Figure 6:**
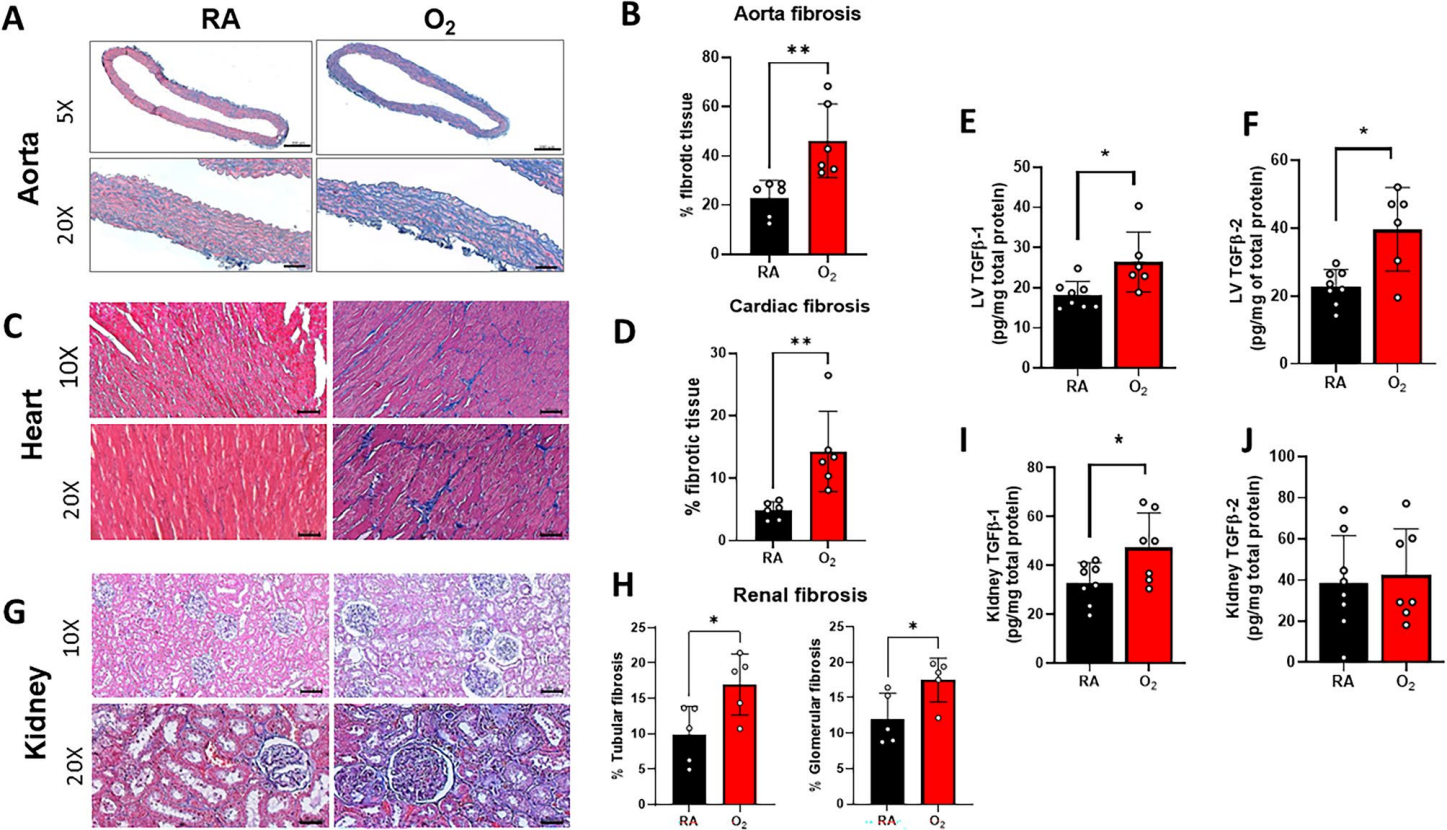
Neonatal hyperoxia increases markers of fibrosis in left ventricle and kidney of adult rats. Masson’s Trichrome staining and fibrosis quantification of **(A, B)** aorta (upper panels, scale bar = 200 µm, 5X magnification, lower panels, scale bar = 50 µm, 20× magnification) **(C, D)** heart (upper panels, scale bar = 100 µm, 10X magnification, lower panels, scale bar = 50 µm, 20× magnification) and (**G, H**) kidney sections (upper panels, scale bar = 100 µm, 10× magnification, lower panels, scale bar = 50 µm, 20× magnification) demonstrating increased fibrosis in the hyperoxia exposed rats by qualitative collagen staining (blue) as well as by quantitative densiometric analysis using ImageJ. n = 5–6/group. Multiplex protein analysis of tissue homogenate showed increased (**E**) TGF-β1 and (**F**) TGF-β2 in the left ventricle and increased (**I**) TGF-β1 and no difference in (**J**) TGF-β2 in the kidney of adult rats exposed to neonatal hyperoxia. n = 6–8/group. Data are mean ± SD; *p < 0.05, **p < 0.01 by unpaired, 2-tailed Student’s t-test; RA = room air; O_2_ = hyperoxia.

Since fibrosis promotes vascular stiffness and cardio-renal dysfunction, we assessed histologic markers of fibrosis and pro-fibrotic protein expression in the heart, aorta, and kidney of adult rats. The densitometric quantitative analysis using Image J software of Masson’s Trichrome stain revealed a significant increase in fibrosis in the aorta (Fig. 6A, B), heart (Fig. 6C, D), and kidney (in both the glomerular and tubular compartments) (Fig. 6G, H) of 1-year-old rats exposed to neonatal hyperoxia. TGF-β1 is a master regulator of fibrosis, implicated in cardio-renal fibrogenesis promoting organ fibrosis through fibroblast proliferation, collagen synthesis and ECM remodeling^18^. Moreover, hyperoxia-exposed adult rats had increased protein expression of TGF-β1 and TGF-β2 in the left ventricle (Fig. 6E, F) and increased TGF-β1 in the kidney (Fig. 6I, J). Taken together, these findings suggest that fibrosis plays a vital role in cardio-renal remodeling after neonatal hyperoxia exposure.

## Discussion

Adults born preterm have an increased risk of vascular stiffness, renal and cardiovascular morbidities^19–21^. Premature infants are routinely exposed to hyperoxia, which is known to contribute to the developmental programming of these adult-onset diseases^7^. Our current study demonstrates that neonatal hyperoxia exposure is associated with increased vascular stiffness in the aorta and mesenteric artery in adult rats accompanied by cardiomyocyte hypertrophy, glomerulomegaly and tubular injury. In addition, there were long-term gene transcriptional changes both in the aorta and kidney, which may be a result of modulation of gene expression induced by the early hyperoxia exposure. These findings were accompanied by increased TGF-β1 expression and associated fibrosis in the left ventricle, aorta, and kidney. To our knowledge, the current study is the first to evaluate vascular stiffness, renal injury, and cardio-renal fibrosis including transcriptional changes following neonatal hyperoxia exposure in a rat model at 1 year of age. These findings have important implications suggesting that prolonged hyperoxia exposure induces long-term cardio-renal dysfunction in preterm survivors by altering gene expression profiles that favor fibrosis.

The neonatal hyperoxia rat model is a well-recognized model of several preterm birth-related conditions^22–24^. Exposure to hyperoxia in the immediate postnatal period in rodents is an established model of bronchopulmonary dysplasia, a condition unique to preterm-born infants^25^. Rodents, unlike humans, undergo postnatal organogenesis for approximately 10–14 days during normal development^26^. Human preterm birth occurs between 22 and 36 weeks of gestation during a period of active organogenesis that is ongoing, albeit for a finite period in the postnatal environment^27,28^. Hence, rodent models provide the ability to test the impact of hyperoxia during ongoing renal and cardiovascular development as experienced by preterm infants. Considering the developmental programming of disease construct has linked hypertension, vascular dysfunction, and impaired renal function in adults born preterm, this experimental model has been utilized to examine the long-term consequences of neonatal oxidative injury of preterm born individuals^11^.

Neonatal hyperoxia has been shown to induce aortic biomechanical changes and cardiac dysfunction in juvenile rats at 6 weeks of age^29^. Here we extended the rodent model to 1 year, roughly equivalent to 35 human years^30^, to study the long-term effects of neonatal hyperoxia exposure and its impact on the systemic vasculature, heart, and kidney. Vascular dysfunction is a well-recognized consequence of preterm birth with effects into adulthood^31–33^. Preterm birth may disrupt or prematurely arrest the development of the vasculature, impacting the vessel structure^31^. Here, we examined the biomechanical alterations both in a large elastic artery, aorta, and in a small resistance artery, mesenteric artery. Pressure myography revealed that rats exposed to neonatal hyperoxia had decreased change from baseline mesenteric lumen diameter as pressure increased. This decrease in mesenteric artery distensibility in hyperoxia-exposed vessels is likely due to alterations in ECM composition rather than changes in medial wall thickness, as there was no change in media-to-lumen ratio or wall thickness compared to RA vessels. In addition, wire myography of the aortic artery also confirmed the increased stiffness and vessel fragility in the hyperoxia-exposed rats. The findings of our current study corroborate those of Mivelaz et al. who demonstrated increased pulse wave velocity in 6 and 9 month-old-rats exposed to 80% hyperoxia from postnatal day 3–10^34^. Importantly, the changes in vessel mechanical properties we observed persisted after a yearlong recovery in RA.

We further investigated the potential mechanism for this vascular stiffness by studying the long-term signaling effects of hyperoxia on the aorta. Neonatal hyperoxia induced persistent transcriptional signaling controlling the connective tissue and extracellular matrix in the aorta, such as “collagen fibril organization”, “ECM organization”, “elastic fiber assembly”, and “aorta development”. The vessel wall is comprised of ECM proteins, including collagen, elastin, glycoproteins, and proteoglycans^32^. Arterial wall stiffness is regulated by factors inherent to the structural composition of the wall, including collagen fibers, elastin fibers, and smooth muscle cells. With the aging of conduit arteries such as the aorta, there is an increase in vascular stiffness due to the alterations in the ECM structure leading to an imbalance between collagen and elastin^32,35^. When thinning and fragmentation of the elastin laminae occur, the mechanical load is transferred to collagen fibers, which are 100–1000 times stiffer than elastic fibers^35^. In the current study, we also found CD 248 (endosialin) to be most differentially expressed between the neonatal RA and hyperoxia-exposed rats at 1 year. During tissue remodeling, CD 248 is associated with proliferation of stromal cells and migration^36^. The upregulation of pathways involved in collagen fibril formation and ECM organization together with the differential expression of CD 248 and stromal cell proliferation in the hyperoxia-exposed rat aorta, supports the concept that neonatal hyperoxia exposure increases vascular stiffness into adulthood.

We found that hyperoxia exposure during ongoing nephrogenesis was associated with glomerulomegaly, tubular injury, renal fibrosis and increased renal TGF-β1 expression in adult rats. In humans, nephrogenesis is typically complete by 36 weeks of gestation; hence, preterm infants born between 22 and 37 weeks gestational age are born with a nephron deficit^19^. Postnatal nephrogenesis in humans occurs for a limited duration and results in the formation of aberrant nephrons^27,37^. In experimental models, hyperoxia is known to limit the potential for normal postnatal nephron formation^7^ and has been shown to induce glomerular and tubular injury in the short term^8,13,38–40^. Jiang et al. showed that neonatal hyperoxia exposure (95% for 1 week followed by 2 weeks of 60%) resulted in short-term renal fibrosis in rats at postnatal day 7 and 21 and was associated with increased total collagen content^39^. Yzydorczyk et al. found that neonatal hyperoxia led to increased blood pressure, vascular dysfunction, microvascular rarefaction, and a 25% reduction of nephron number in adult rats aged 15–35 weeks^11^. However, similar to our study, other studies have not found any significant differences in nephron number^13,14,38^. The inconsistent findings related to outcomes after hyperoxia exposure during nephrogenesis may be secondary to differences in hyperoxia concentration and duration. In addition, it is possible that the mechanism of glomerulomegaly as a sign of injury is independent of decreased nephron number but still represents a state of hyperfiltration, which is associated with long-term risk of chronic kidney disease^41,42^. Urine biomarkers have been studied in preterm infants as an early indication of kidney dysfunction^43,44^. Urine biomarker profiles follow developmental changes with advancing chronological and postmenstrual age that are important when distinguishing kidney injury among preterm infants^43^. In this study, urine albumin and NGAL trended higher in the 1-year rats exposed to neonatal hyperoxia, which is consistent with other studies, however this was not statistically significant.

We found that hyperoxia exposure during nephrogenesis was associated with increased renal fibrosis markers in adulthood, including elevated TGF-β1 kidney expression, upregulation of MMP-7 and MMP-9 and downregulation of genes involved in the antifibrotic BMP signaling pathway. MMPs are proteolytic enzymes involved in ECM deposition, inflammation, vascular damage, and apoptosis^45,46^. MMP-7 and MMP-9 are associated with a profibrotic effect and are protective against renal fibrosis after unilateral ureteral obstruction^47^. Neonatal hyperoxia exposure also downregulated, BMP signaling at 1 year. BMP-7 is highly expressed in the kidney and is essential for kidney development^48,49^. There was a persistent downregulation of key kidney development genes, including PAX2 and WNT9B, in adult rats after neonatal hyperoxia exposure. Cwiek et al. showed that mice delivered preterm under normoxic conditions underwent premature cessation of nephrogenesis, resulting in a lower glomerular density and premature differentiation of nephron progenitor cells^50^. Moreover, reduced PAX2 levels were evidenced in a unilateral nephrectomy rat model during nephrogenesis^51^. It is possible that hyperoxia exposure during nephrogenesis further impairs nephron progenitor differentiation by downregulating genes that are critical for kidney development. Importantly, the alterations in renal developmental genes induced by the hyperoxia exposure seem to have persistent implications into adulthood, lending towards renal injury and fibrosis.

Clinical studies have demonstrated altered cardiac structure, including hypertrophy and cardiac dysfunction in adults born preterm^52–54^. These reported increases in LV mass in adults born preterm is concerning given that LV hypertrophy is a major independent predictor for cardiovascular disease^55,56^. Consistent with these clinical studies, our study has shown evidence of cardiomyocyte hypertrophy in adult rats after neonatal hyperoxia exposure.

Fibrosis is a unifying factor in the cardio-renal pathology continuum and is a consequence of inflammation and oxidative stress-related endothelial dysfunction in aging, hypertension, and ischemia^57^. Clinical studies in young adults born preterm using cardiac MRI and echocardiography have shown that prematurity is associated with myocardial fibrosis ^58,59^. Our current study defines a parallel increase in fibrosis in the heart, aorta, and kidney in adult rats exposed to neonatal hyperoxia. Here we confirm previous reports in rodent models that show early postnatal hyperoxia exposure leads to cardio-renal fibrosis^9,12,29^. Neonatal hyperoxia exposure is known to induce oxidative stress^7^. This stress could be accentuated in the setting of suboptimal antioxidant defenses in preterm infants. Interestingly, in the current study the kidney RNA-sequencing analysis, showed upregulation of glutathione gene expression, an antioxidant marker, in the adult rats exposed to neonatal hyperoxia. It is known that renal redox signaling during the transition from acute to chronic kidney injury is a complex and dynamic process in which both oxidative and antioxidative mechanisms are activated^60^. The evolution of markers of oxidative stress and antioxidant capacity from the neonatal to adult stage requires future investigation which was beyond the scope of the current study. While the exact pathogenesis of vascular stiffness and renal impairment seen in adults exposed to neonatal hyperoxia is unclear, with increased release of reactive oxygen species, proapoptotic pathways are activated and inflammatory cells are released^61^. Activated inflammatory cells release proinflammatory cytokines and growth factors which stimulate fibroblast proliferation and matrix remodeling. Together these events are speculated to give rise to neonatal hyperoxia-induced vascular stiffness and renal impairment^62^.

The strengths of the current study include the long-term follow up of the animals for 1 year. In addition, our study is strengthened by using both large elastic and small resistance arteries in assessments of vessel integrity and mechanical properties. Moreover, this is the first report of differential gene expression profiles in adult vasculature and kidney after neonatal hyperoxia exposure, which may provide important information to guide the development of therapeutic targets to reduce the burden of cardiovascular and renal dysfunction after preterm birth. Our study, however, has certain limitations. The phenotype observed in the neonatal hyperoxia rat model is severe and may amplify the disease evidenced in premature infants. The mortality in the hyperoxia group was high and we speculate that this was related to either cardiovascular or chronic lung disease as suggested by both experimental and clinical studies^9,24,29,63^. We however chose 2 weeks of oxygen exposure in neonatal rats as it represents an equivalent stage of human development from 24 to 38 weeks gestational age and would represent an infant population needing prolonged oxygen support^64^. In addition, the neonatal hyperoxia model does not fully encompass the multiple maternal and neonatal risk factors associated with prematurity and may not comprehensively reflect the disease evidenced in many preterm survivors. We were not able to assess the blood pressure, heart weight, or renal function in these 1-year-old rats. We were unable to evaluate sex differences due to the small sample size. In addition, because we sought to evaluate long term sequelae of neonatal hyperoxia exposure we did not assess our outcomes in younger rats as this was outside the scope of the current study. Future studies to investigate the impact of hyperoxia exposure on short- and long-term outcomes related to oxidative stress signaling and its relationship with the development of hypertension, cardio-renal fibrosis, and renovascular remodeling will be important.

In conclusion, our study demonstrates the commonality of matrix remodeling and fibrosis in the aorta, heart, and kidney and more importantly, reveals key etiology-specific signatures in adult rats exposed to neonatal hyperoxia. These findings suggest that transcriptome signatures may distinguish vascular stiffness, shedding light on underlying biological changes after neonatal hyperoxia-induced vascular remodeling. We also provide further evidence for the long-term relevance of early life events on developmental programming of the cardio-renal systems. We have elaborated on our knowledge of the contributors to vascular stiffness and cardio-renal dysfunction in survivors of preterm birth who were exposed to hyperoxia therapy in the neonatal period. Though the impact of adverse perinatal environmental exposures may not be evident until additional risk factors are introduced during the lifespan, understanding the long-term consequences of neonatal hyperoxia exposure could provide novel approaches to the development of interventional strategies to mitigate the burden of cardio-renal dysfunction after preterm birth.

## Materials and methods

### Ethics statement

The protocol was approved by the Animal Care and Use Committee at the University of Miami Miller School of Medicine (#17-136). We performed this study in strict accordance with the recommendations in the Guide for the Care and Use of Laboratory Animals of the National Institutes of Health. The study is reported in accordance with the ARRIVE guidelines. All surgery was performed under isoflurane anesthesia, and every effort was made to minimize suffering.

### Experimental model

Pregnant Sprague–Dawley rats were obtained from Charles River Laboratories (Wilmington, MA) and were housed with food and water available ad libitum at constant temperature (25° C) under 12:12 light/dark cycle. Rat pups (N = 26, postnatal day 1) were randomized to room air (RA; n = 13) or hyperoxia (85% O_2_; n = 13) for 2 weeks. They were then recovered in RA until they were 1-year-old. During the hyperoxia exposure, the pups were housed in a plexiglass chamber with continuous hyperoxia exposure and monitoring that was briefly interrupted for animal care (< 10 min/day). Mothers were rotated every 48 h between RA and hyperoxia chambers to prevent lung damage. At the time of sacrifice, body weight and kidney weight were recorded.

### Ex-vivo mesenteric artery stiffness- pressure myography

Mesenteric artery was isolated from 1-year-old rats via microdissection. Mesenteric arteries were evaluated via pressure myography by person blinded to the groups. Arteries were mounted on glass pipet tips in microvessel perfusion chambers as previously reported^65,66^. Chambers were placed on inverted microscopes equipped with a digital image capture system (IonOptix) to measure the inner lumen diameter and outer vessel wall diameter. Vessels were warmed to 37 °C, pressurized to 5 mmHg using a column of Krebs buffer, and allowed to equilibrate (30 min). Intraluminal pressure was increased by raising the column of Krebs buffer in a stepwise manner. Changes in diameter were allowed to plateau (20 min) before pressure was increased. Average vessel wall thickness was calculated by dividing the difference of the inner and outer diameters by 2. Krebs buffer recipe used was 109 mM NaCl, 4.7 mM KCl, 2.5 mM CaCl_2_, 0.9 mM MgSO_4_, 1 mM KH_2_PO_4_, 11.1 mM glucose, and 34 mM NaHCO_3_; titrated to pH 7.3.

### Ex-vivo aortic stiffness- wire myography

The abdominal aorta was used for stress testing using a Radnoti organ bath system as previously reported^67^. Arteries were suspended on stainless steel triangular hooks, one of which was connected to a force transducer and mechanical stepper motor while the other was anchored to a glass rod at the base of the tissue bath. Vessels were submerged in Krebs buffer warmed to 37 °C and placed under 0.5 g tension for 30 min to equilibrate. The tension (g) in each vessel sample was measured continuously using PowerLab/8 SP (ADInstruments) hardware and analyzed with LabChart 7 Pro software (ADInstruments). Every four minutes, the stepper motor rotated 0.1 mm/s for a total of 10 s (1 mm displacement) resulting in the hooks being pulled apart. This protocol was repeated until tissue failure. The number of stepper motor turns required to reach tissue failure was recorded. The impulse stiffness (stress immediately after stepper motor rotation) and equilibrium stiffness (stress immediately prior to the next motor rotation) was calculated using the following formulas:

$${\text{Stress}}\left( {{\text{Pa}}} \right)\, = \,{\text{force}}/{\text{area}}$$

$${\text{force }} = {\text{ measured}}\;{\text{tension}}*{\text{acceleration}}\;{\text{of}}\;{\text{gravity}}$$

$${\text{area}} = {\text{the}}\;{\text{length}}\;{\text{of}}\;{\text{the}}\;{\text{vessel}}*{\text{wall}}\;{\text{thickness}}*{2}$$

### Renal histology

Kidneys were harvested at 1 year, fixed in formaldehyde and paraffin-embedded. Serial 5 µm sections (in the coronal plane) were stained with hematoxylin and eosin (H&E) and examined by light microscopy. Multiple digital microphotographs (10–15) were taken for each section at 2.5× and 40× magnification to assess glomerular area and tubular injury scoring^39^. Glomerular number was estimated by counting the total number of cortical glomeruli per high power field (hpf; 5X) from 10 sections of a longitudinal cross-section per animal and averages reported.

### Glomerular measures

In the 40× field of view, ten mature glomeruli were selected at random for histomorphometry. Using Image J analysis software, the boundary of the Bowman’s capsule of each glomerulus (10 per kidney) and the average glomerular cross-sectional area were determined by a nephrologist blinded to the treatment group and trained on use of the software program as previously described.

### Tubular injury score

A pathologist blinded to the treatment group examined at least 40 cortical fields at 100× magnification. Tubular injury was defined by any of the following features: tubular dilatation, tubular atrophy, vacuolization, degeneration and sloughing of tubular epithelial cells, or thickening of tubular basement membrane. The scoring system used was as follows: 0 = no tubular injury; 1 ≤ 10% of tubules injured; 2 = 10–25% of tubules injured; 3 = 26–50% of tubules injured; 4 = 51–75% of tubules injured; and 5 ≥ 75% of tubules injured^68^.

### Cardiomyocyte hypertrophy

Heart tissues were fixed in 4% formaldehyde embedded in paraffin. Several sections at 5 µm interval were obtained for histology. Heart slides stained with H&E were used to measure cardiomyocyte cross sectional area (CSA) in the LV zone. Briefly, transverse sections of the heart were measured randomly in 6–7 hpf. We measured the area of approximately 200 cardiomyocytes per heart section.

### Masson’s trichrome staining

Paraffin-embedded sections of the abdominal aorta, heart and kidney were deparaffinized, rehydrated and stained with Masson’s Trichrome as per manufacturer’s instructions (Cat# HT15-1KT, Sigma-Aldrich). Fibrosis was qualitatively determined in a blinded fashion using a light microscope at 40X magnification. Densitometric quantitative analysis using Image J software was obtained in the distal abdominal aorta, left ventricle, and kidney^69^. In the kidney, glomerular and tubular sclerosis scores were calculated separately.

### Urine biomarker analysis

Urine was collected by suprapubic aspiration at the time of sacrifice. Urine samples were frozen at − 80° C and stored until processing. Urine was outsourced to Eve Technologies (Calgary, AB, Canada) for the Rat Kidney Toxicity Panel 2 3-Plex Assay, a multiplex immunoassay, which included the following candidate biomarkers: albumin, cystatin-C, and NGAL. Absolute levels were converted to nanograms (ng)/ml for cystatin C and NGAL and to micrograms (µg)/ml for albumin.

### RNA isolation and sequencing

Total RNA was extracted from frozen aortic arch and thoracic aorta and kidney tissues using the RNeasy Universal Mini Kit (Cat#217004; Qiagen Inc, Valencia, CA) according to the manufacturer’s instructions. RNA quality and integrity were verified using the Agilent 2100 Bioanalyzer (Agilent, Agilent Technologies, Santa Clara, CA). All samples had RNA integrity number > 7. RNA sequencing was performed by BGI Genomics with a read depth of 30 million reads per sample for 150 bp paired-end reads. The raw sequence reads in FASTQ format were aligned to the rat (Rattus norvegicus) genome build rn6.0 using kallisto^70^ followed by gene summarization with tximport^71^. After checking data quality, differential expression analyses were performed using DESeq2^72^. Genes were considered differentially expressed based on their *p*-value (< 0.05) and FDR (Benjamin-Hochberg, < 0.1). Lists of differentially expressed genes were used for functional enrichment analysis of Gene Ontology terms and Pathways was performed on ToppCluster^73^. RNA-sequencing was performed on samples from 4 animals per group for each organ. One hyperoxia exposed kidney was removed from the analysis as it was identified as an outlier on Principal component analysis (PCA) analysis.

### Real-time qRT-PCR

Total RNAs isolated as above were reverse transcribed (Superscript VIVILO Master Mix; Cat# 11766050, ThermoFisher, Cambridge, MA). Real-time qRT-PCR using gene-specific primers and TaqMan Fast Advanced Master Mix (Cat #4444554, Applied Biosystems, Foster360 City, CA) was performed on an ABI Fast 7500 system (Applied Biosystems) as previously described^74^. Table 1 lists the rat primers used. The expression levels of target genes were normalized to GAPDH (Cat#Rn99999916_s1, ThermoFisher).

**Table 1:** Primer sequences for PCR amplification.

Name	Sequence (5'—> 3')	Length
Arntl	F: CTATCTTCCTCGGACACCGC	20
	R: GAACCATGTGTGAGTGCAGC	20
CD248	F: AGCCGGTCCAGCTATCTACA	20
	R: ATCTGCTGCTAGACGGAAGC	20
COL3A1	F: AGGGAACAA CTG ATG GTG CTA CTG	20
	R: GGACTGCTGTGCCAA AATAAGAGA	20
Yme1l1	F: AGGCGTTGCTGACCTATGAG	20
	R: CAGAAGTGGGGGTTGGAAGT	20
MMP7	F: AAGTTCTTCGGTTTGCCGGA	20
	R: TTGTCTCCGTGATCTCCCCT	20
PAX2	F: GATCCGGGACAGGCTGCTA	19
	R: GAGTGGTGCTCGCCATATCA	20
Cyp8b1	F: CTGTGGGGTTCAGTGCTAGG	20
	R: TTCATGGCGTCCTGATGCTT	20
WNT9B	F: AGAATGACTGCTGGTGAGCC	20
	R: GTCAGCCTCAGCCTCAACTT	20
BNP	F: AGCTCTCAAAGGACCAAGGC	20
	R: TCCGGTCTATCTTCTGCCCA	20
ANF	F: GGCACTTAGCTCCCTCTCTG	20
	R: AATGCGACCAAGCTGTGTGA	20

### Multiplex protein quantification

Flash frozen left ventricle and kidney tissues were homogenized in RIPA buffer (Cat# sc-24948, Santa Cruz Biotechnology, Santa Cruz, CA) and centrifuged at 15,000 rpm for 15 min at 4 °C. The protein concentration of the supernatant was measured by BCA protein assay (Cat# PI2322 ThermoFisher Scientific, Waltham, MA). All the samples were then diluted for target protein concentration of 100 mg/ml. These protein samples (100 ml) were outsourced to Eve Technologies (Calgary, AB, Canada) for measuring TGF-β1-3 protein concentrations using the Rat TGF-β 3-Plex Discovery Assay, a multiplex immunoassay^75^. TGF-β concentration was expressed as pg/mg of total protein.

### Statistics

Normality was assessed with the Shapiro–Wilk test. Based on the distribution of the data, an unpaired, 2-tailed Student’s t-test was used to determine significance. For pressure myography, one-way ANOVA with Bonferroni’s correction for multiple comparisons was used to evaluate differences among groups. Data are expressed as mean ± SD. A *p* < 0.05 was considered significant. All analyses were performed using commercially available statistical software packages and graphs generated in GraphPad Prism version 9.4.0 for Windows, (GraphPad Software, San Diego, CA).

## Data Availability

Sequence data that support the findings of this study have been deposited in Gene Expression Omnibus, GEO accession GSE234635.
